# 707. Molecular Epidemiology of *Clostridioides difficile* Colonization in Families with Infants

**DOI:** 10.1093/ofid/ofad500.769

**Published:** 2023-11-27

**Authors:** Philip Toltzis, Christine Marlow, Jason Clayton, Gregory Golonka, Lynn Maruskin, Annette Jencson, Hosoon Choi, Piyali Chatterjee, Munok Whang, Chetan Jinadatha, Jennifer Cadnum, Curtis Donskey

**Affiliations:** Rainbow Babies and Children's Hospital, Cleveland, Ohio; Rainbow Babies and Children's Hospital, Cleveland, Ohio; Rainbow Babies and Children's Hospital, Cleveland, Ohio; Kids in the Sun, Strongsville, Ohio; Kids in the Sun, Strongsville, Ohio; Cleveland VA Hospital, Cleveland, Ohio; Central Texas Veterans Health Care System, Temple, Texas; Central Texas Veterans Health Care System, Temple, Texas; Central Texas Veterans Health Care System, Temple, Texas; Central Texas Veterans Health Care System, Temple, Texas; Louis Stokes Cleveland VA Medical Center, Cleveland, Ohio; Cleveland VA Hospital, Cleveland, Ohio

## Abstract

**Background:**

We previously reported a high incidence of *C. difficile* colonization among parents caring for an asymptomatically-excreting infant (IDWeek 2022, Abs 2007), suggesting infants as a nidus of adult infection. Herein we define the molecular epidemiology of the organisms isolated within these families.

**Methods:**

Families were enrolled at the baby’s 4^-^month well child visit at a suburban pediatric practice. Soiled diapers were mailed every two weeks for 4 months (total 8 time-point (TPs)) to a research laboratory. Stool was inoculated onto CDBA plates with portions broth- enriched. Simultaneously sent parental diaper wipes were handled identically. *C. difficile* was identified by colony morphology and GDH assay. Specimens from each family were assessed for relatedness by PCR ribotyping, and Whole Genome Sequencing (WGS) was utilized to determine genetic relatedness of repeatedly excreted organisms over time, both employing methods previously described (ICHE 2021;42:731).

**Results:**

30 families (babies and mothers plus 22 fathers) participated, with 25 families submitting specimens at > 4 TPs. Prevalence of infant colonization/TP ranged from 50-72%, and cumulatively 90% were positive at least once (median 4 samples positive/subject). Prevalence among mothers was 18-39%/TP (cumulative 87%, median 2 samples positive/subject) and fathers 20-41%/TP (cumulative 86% positive, median 1 sample positive/subject). In 27 families *C. difficile* was isolated from > 1 member, with all but 1 including the infant. Isolates from 24 families could be ribotyped. In 6 families each member had a non-identical ribotype, in 3 families there was a single identical ribotype, and in 15 families some ribotypes were shared but > 1 member harbored > 1 ribotype. For example, one infant-mother pair shared two different ribotypes, but each harbored 1 additional distinct ribotype. WGS of persistently carried isolates with identical ribotypes from both infants and adults were related, usually exhibiting < 3 SNP differences.

**Conclusion:**

*C difficile* is prevalent in families caring for infants. Molecular analysis revealed a previously unrecognized complex epidemiology, with most family members sharing at least 1 common strain but frequently accompanied by additional unrelated ribotypes.

**Disclosures:**

**All Authors**: No reported disclosures

